# DKK1 in Cancer: A Bench-to-Bedside Review of Molecular Mechanisms and Clinical Applications

**DOI:** 10.3390/cancers18091375

**Published:** 2026-04-25

**Authors:** Meiheng Sun, Yuzhe Wang, Yihao Zhang, Ziqi Chen, Yuanyuan Yu, Aiping Lu, Wei Kang, Qianjun Chen, Ge Zhang, Jianhui Tian, Bao-Ting Zhang

**Affiliations:** 1 School of Chinese Medicine, The Chinese University of Hong Kong, Hong Kong, China; 2 Department of Oncology, Shanghai Municipal Hospital of Chinese Medicine, Shanghai University of Traditional Chinese Medicine, Shanghai 200071, China; 3 Law Sau Fai Institute for Advancing Translational Medicine in Bone & Joint Diseases, Hong Kong Baptist University, Hong Kong, China; 4 Department of Anatomical and Cellular Pathology, State Key Laboratory of Translational Oncology, Sir Y.K. Pao Cancer Center, Prince of Wales Hospital, The Chinese University of Hong Kong, Hong Kong, China; 5 Department of Breast, Guangdong Provincial Hospital of Chinese Medicine, Guangzhou 510120, China; 6 State Key Laboratory of Traditional Chinese Medicine Syndrome, The Second Affiliated Hospital of Guangzhou University of Chinese Medicine, Guangzhou 510120, China; 7 Chinese Medicine Guangdong Laboratory, Guangzhou 510006, China

**Keywords:** cancer, Dickkopf-1, mechanism, targeted therapy, clinical trails

## Abstract

Dickkopf-1 (DKK1) is a secreted glycoprotein that traditionally acts as an antagonist of canonical Wnt/β-catenin signaling. Although it functions as a tumor suppressor in some specific biological background and disease stages, growing evidence links DKK1 to tumor progression, immune evasion, and therapy resistance in a variety of multiple malignancies. This review provides a comprehensive bench-to-bedside overview of DKK1 in cancer. We first delineate how DKK1 regulates both Wnt-dependent and Wnt-independent pathways. From a clinical perspective, we evaluate the application potential of DKK1 as a diagnostic and prognostic biomarker. We further discuss the progress of DKK1-targeted interventions, ranging from monoclonal antibodies in clinical trials to next-generation therapeutic modalities. Finally, we discuss the challenges in clinical translation and suggest future directions for DKK1-based precision medicine. In summary, by integrating preclinical insights with current clinical data, this review provides a strategic roadmap for advancing DKK1-targeted therapies in cancer.

## Simple Summary

Dickkopf-1 (DKK1) is a protein that plays a complex role in cancer. Traditionally, it was regarded as a tumor suppressor due to its inhibitory effects on Wnt signaling. However, emerging evidence suggests its important role in promoting tumor progression, helping cancer cells escape the immune system attacks, and inducing therapeutic resistance. This review summarizes the complex biological mechanisms of DKK1 and its application potential as a diagnostic tool and a therapeutic target in cancer. We also discuss the progress of emerging DKK1-targeted therapies, such as monoclonal antibodies and next-generation modalities, in preclinical and ongoing clinical trials. Finally, we outline key future priorities to advance DKK1-based precision medicine in cancer.

## 1. Introduction

The Dickkopf (DKK) family consists of four secretory glycoproteins (DKK1-4) and a distinct DKK3-related protein (DKKL1). DKK1 was traditionally regarded as a crucial regulator of head formation during Xenopus embryonic development [1], but it has since been recognized as a key regulator in adult tissue homeostasis, particularly in bone remodeling [2]. Structurally, DKK1, DKK2, and DKK4 are characterized by two highly conserved cysteine-rich domains (CRD1 and CRD2), whereas DKK3 is more structurally divergent and often functions independently of the Wnt signaling pathway [3,4].

DKK1 is the most extensively characterized inhibitor of the canonical Wnt/β-catenin signaling pathway. It functions by binding to the Wnt co-receptors, low-density lipoprotein receptor-related protein 5/6 (LRP5/6), and the transmembrane protein Kremen1/2 [5,6]. Then, this ternary complex formation triggers the endocytosis of LRP5/6, preventing the stabilization of β-catenin and the downstream gene transcription [7]. While this antagonism is important for normal physiology, its dysregulation is a hallmark of many cancers.

The role of DKK1 in oncology presents a complex biological paradox. In early-stage colorectal cancer (CRC) and papillary thyroid carcinoma (PTC), it was reported that DKK1 was often down-regulated due to high hypermethylation of the promoter, indicating its traditional anti-tumor role [8]. However, emerging clinical data has revealed the different expression of DKK1 in advanced malignant tumors, including multiple myeloma (MM), hepatocellular carcinoma (HCC), gastric cancer (GC), non-small-cell lung cancer (NSCLC), etc. [9,10,11]. In these cases, elevated DKK1 levels in serum and tissues were reported and were correlated with advanced tumor stage, lymph node metastasis, and poor overall survival (OS) [12,13,14].

The discovery of the Wnt-independent signaling axis that mediated by DKK1 is the key mechanism to explain this functional shift. For instance, the identification of Cytoskeleton-associated protein 4 (CKAP4), a high-affinity receptor for DKK1, elucidated a direct mechanism by which DKK1 promotes tumor proliferation regardless of Wnt activity [15]. Furthermore, recent research suggested that DKK1 could also serve as a key regulator of the tumor immune microenvironment (TIME). For instance, by recruiting myeloid-derived suppressor cells (MDSCs) and inhibiting natural killer (NK) cell activity, DKK1 was reported to facilitate immune evasion and mediates resistance to immune checkpoint inhibitors (ICIs) [10,14,16].

Although the first-generation DKK1 inhibitors, such as DKN-01, have shown certain therapeutic promise in specific subgroups, achieving a broad and stable anti-tumor efficacy is still a clinical challenge. Therefore, we need to have a deeper understanding of this target in cancer. This review aims to summarize the updated knowledge of DKK1, including its application value as biomarker and mechanism of action. We also discuss the gap between molecular research and clinical outcomes. We then summarize the latest findings of ongoing preclinical and clinical trials of monoclonal antibodies and next-generation candidate drugs. We aim to provide a future direction for DKK1-based precision medicine to improve the clinical therapeutic effect in cancer.

## 2. DKK1 Expression in Human Cancers

### 2.1. Pan-Cancer Expression Landscape

Large-scale studies, including data from The Cancer Genome Atlas (TCGA), show that DKK1 expression is highly heterogeneous across different cancers [17]. While DKK1 is low or undetectable in most healthy adult tissues, its reactivation is usually a hallmark of aggressive malignancies [18]. DKK1 expression seems to be not constant but shifting during development. And its clinical impact depends on the specific type of cancer (Table 1).

#### 2.1.1. Oncogenic Overexpression

In the majority of solid tumors, DKK1 functions as a prominent oncoprotein. It was reported that the elevated expression of DKK1 was frequently associated with increased tumor aggressiveness, therapeutic resistance, and immunosuppression.

In the digestive system, DKK1 is a well-established marker in GC and esophageal carcinoma. It was reported that DKK1 was overexpressed in tumor tissues compared to adjacent normal tissues [20]. And high expression of DKK1 was correlated with lymph node metastasis and advanced TNM stages [35,36]. In the hepatobiliary–pancreatic axis, it was reported that the expression of DKK1 was upregulated in HCC tissues and serum, particularly in patients with high-grade tumors and vascular invasion [9,22]. Similarly, the upregulation of DKK1 could serve as a prognostic marker in cholangiocarcinoma [23] and pancreatic adenocarcinoma [21].

Regarding thoracic and head and neck malignancies, DKK1 was reported to play a critical role in tumor differentiation and microenvironment modulation. In NSCLC, encompassing both lung adenocarcinoma [19] and lung squamous cell carcinoma [18], DKK1 overexpression was reported to be not only associated with poor prognosis but also linked to poor differentiation and acquired resistance to EGFR-TKIs through cancer-associated fibroblast (CAF) interactions [37]. Similarly, in head and neck squamous cell carcinoma (HNSC), DKK1 overexpression could serve as an indicator of an immunosuppressive TME [11,38].

In the genitourinary and gynecological spectrum, elevated DKK1 expression was reported to correlate with poor prognosis in triple-negative breast cancer (TNBC) [24,39], ovarian cancer [28], and cervical cancer [27]. Notably, in TNBC, high DKK1 level is also an indicator for immunosuppression. Furthermore, DKK1 overexpression was reported to be associated with aggressiveness in genitourinary cancers, including prostate cancer [25] and urothelial carcinoma [30].

Finally, in addition to solid tumors, DKK1 also plays an important role in malignant tumors of the blood system, especially in MM, where it acts as a key mediator of bone disease and tumor load [26].

#### 2.1.2. Contextual Loss/Silencing

Under specific biological background and tumor stage, DKK1 retains its function as a tumor suppressor. In early-stage CRC, DKK1 was reported to be often downregulated due to promoter hypermethylation [8]. The lack of DKK1 expression may lead to overactivation of Wnt signaling, thus promoting early formation of tumors.

In addition, atypical subcellular translocation and exogenous secretion from other cells redefine its clinical significance. For example, as a non-canonical driving factor for chemoresistance, the nuclear localization of DKK1 marks the transformation of its mechanism of action from traditional secretory inhibitor to other novel mechanisms of action [33]. Furthermore, CAFs within the TME could secrete DKK1, thus promoting the survival of tumor cells [40]. Paradoxically, the decrease in DKK1 within tumor contrasts with the increase in DKK1 in the circulation, indicating that it is not only a Wnt inhibitor, but also a multifunctional oncogenic regulator [34].

Similar to CRC, DKK1 was reported to be usually downregulated in PTC tissues and was negatively correlated with tumor size [31,41]. In addition, although DKK1 was reported to show a downward trend in endometrial cancer [32], the expression of DKK1 in patients with Wnt-activating mutations was reported to be higher than that of patients with wild-type Wnt signaling [42]. These findings indicate that DKK1 is not a static tumor suppressor, but a dynamic regulator whose role is highly dependent on the environment and disease stage.

### 2.2. Correlation with Clinicopathological Features

#### 2.2.1. Diagnostic Biomarker

The secretory characteristics of DKK1 make it an ideal candidate marker for liquid biopsy. Although alpha-fetoprotein (AFP) is still the standard biomarker for HCC, a considerable number of HCC patients still have negative AFP-test outcomes. Clinical studies showed that serum DKK1 detection could exhibit high sensitivity and specificity for the diagnosis of AFP-negative HCC, which could effectively distinguish between malignant lesions and liver cirrhosis and chronic hepatitis [43]. Furthermore, the combined detection of DKK1 and AFP could greatly improve the diagnostic accuracy of early-stage HCC, indicating DKK1 as a complementary tool for risk stratification [44]. Similar diagnostic value was also observed in GC. The increase in serum DKK1 levels was reported to reflect high tumor load [45]. Beyond serum markers, DKK1 also showed diagnostic utility in body fluids. In malignant mesothelioma (MESO), elevated DKK1 levels in pleural effusions could help to differentiate malignant from non-malignant cases, providing a complementary tool for cytological diagnosis [29].

However, the diagnostic performance metrics reported in the aforementioned studies are primarily derived from individual studies or small-scale cohorts and have not yet been externally validated in independent patient populations across different tumor types. Furthermore, there is significant heterogeneity among studies regarding biological sample types, testing platforms, data preprocessing, and the definition of diagnostic thresholds. Given that these methodological differences may affect comparability across different cohorts, DKK1 is still considered a promising diagnostic biomarker rather than a fully standardized one. Before broader clinical application, follow-up studies employing a unified testing protocol, predefined thresholds, and large, independent validation cohorts are urgently needed.

#### 2.2.2. Prognostic Biomarker

The prognostic value of DKK1 is supported by its strong association with tumor progression and aggressiveness across multiple malignancies.

In the digestive system, DKK1 may serve as a predictor of poor survival. In GC, DKK1 overexpression was reported to be an independent prognostic factor that correlated with depth of invasion, distant metastasis, and shortened OS [35,46]. Similarly, in HCC, high levels of DKK1 in tissue and serum were reported to be associated with vascular invasion and post-operative recurrence [43,47]. This prognostic potential also extends to esophageal adenocarcinoma [20], cholangiocarcinoma [23], and pancreatic adenocarcinoma [21].

In gynecological and genitourinary tumors, the level of DKK1 was reported to be usually associated with advanced disease stages. In prostate cancer, elevated serum level of DKK1 at the time of diagnosis indicates a shorter survival, reflecting a heavy metastatic load [25]. Similar situations related to DKK1 and poor prognosis were also found in ovarian cancer [28], cervical cancer [27], and urothelial carcinoma [30], suggesting its application potential as a biomarker for aggressive phenotypes in the pelvic region.

In thoracic and head and neck cancers, DKK1 upregulation could serve as a marker of therapy resistance and poor differentiation. In HNSC and lung cancer, high DKK1 expression was reported to be associated with reduced survival rates [18,48].

Finally, in hematologic malignancies, particularly MM, DKK1 overexpression was reported to be not only a driver of osteolytic bone disease but also a prognostic factor correlating with poor survival [26].

## 3. Preclinical Mechanisms of DKK1 in Tumor Pathogenesis

### 3.1. Canonical Wnt/β-Catenin-Dependent Mechanisms

#### 3.1.1. Immune Modulation

It was reported that DKK1 could regulate TIME via MDSCs. In HNSC, tumors with high expression of DKK1 showed unique immunosuppressive characteristics, including T cell exclusion and MDSC accumulation [49]. Mechanistically, it was reported that DKK1 could promote immune evasion by inhibiting canonical Wnt/β-catenin signaling within the MDSCs. Blocking the canonical Wnt signaling may be crucial to promote the expansion and immunosuppression of MDSCs, thereby inhibiting the recruitment of T cells and promoting tumor progression [50].

On the other hand, DKK1 was also reported to play a pivotal role in immune evasion from NK-cell-mediated clearance by inhibiting canonical Wnt signaling within tumor cells. By inhibiting autocrine Wnt signaling within cells, DKK1 could induce a state of metastatic latency or quiescence. This mechanism was reported to promote the downregulation of ULBP on tumor cells, a ligand for activating NK cells, thereby shielding them from recognition and elimination by NK cells [51]. These Wnt-dependent mechanisms illustrate how DKK1 coordinates a multi-lineage defense to help malignant cells evade from the immune system.

#### 3.1.2. Inhibiting Tumor Progression and Metastasis

Given that the Wnt signaling pathway could promote tumor progression and metastasis [52], its antagonist, DKK1, was found as a tumor suppressor in specific tissue types and early disease stages. In human PTC, colon cancer, and breast cancer [41,53,54], DKK1 was reported to promote β-catenin phosphorylation and degradation, thereby silencing TCF/LEF-dependent transcription. These findings suggested that DKK1 could exert its anti-tumoral effects via Wnt pathway inhibition, which was further supported by the observation that these phenotypes are reversed in the presence of constitutively active β-catenin.

#### 3.1.3. Therapy Resistance

It was reported that DKK1-mediated Wnt inhibition could also promote therapy resistance. This mechanism was examined in MM, where DKK1 could play a critical role in acquired resistance to bortezomib (BTZ). Studies suggested that DKK1 could promote drug resistance by suppressing the expression of WWP2, an E3 ubiquitin ligase and direct target of Wnt/β-catenin signaling. Mechanistically, DKK1-mediated inhibition of the canonical Wnt axis could lead to the downregulation of WWP2, which in turn prevents the ubiquitination and degradation of GLI2, a key transcription factor in the Hedgehog (Hh) pathway. Consequently, this DKK1-induced stabilization of GLI2 could activate Hh signaling, thereby contributing to BTZ resistance in MM cells. These findings indicate a complex signaling crosstalk where DKK1 can exploit the Wnt-dependent DKK1-WWP2-GLI2 axis to drive therapeutic resistance [55].

**Figure 1:**
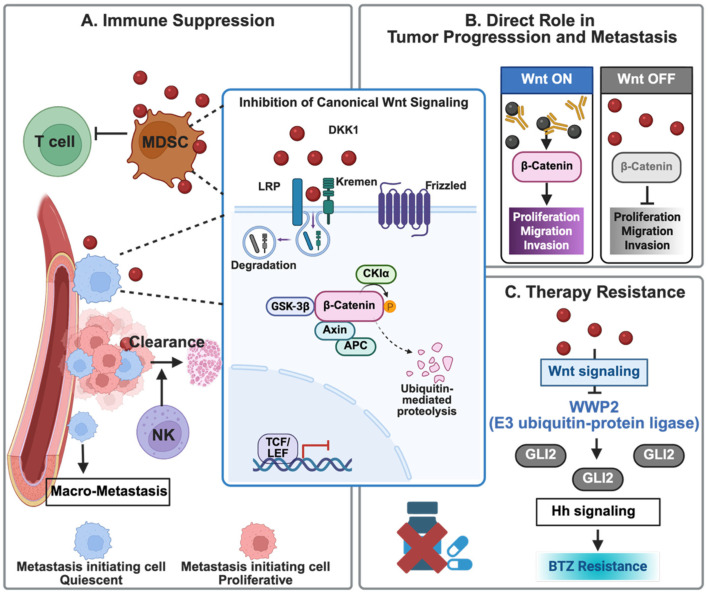
The multifaceted roles of DKK1 in cancer via canonical Wnt-signaling dependent mechanisms. (**A**) Immune Modulation: DKK1 can promote the expansion and immunosuppressive function of MDSCs, leading to T cell exclusion. Furthermore, DKK1 can downregulate stress ligands (e.g., ULBP) on metastasis-initiating tumor cells, facilitating evasion from NK cell-mediated clearance. (**B**) Regulation of Tumor Progression and Metastasis: DKK1 can suppress tumor proliferation, migration, and invasion by promoting the ubiquitin-mediated proteolysis of β-catenin. (**C**) Mechanisms of Therapy Resistance: DKK1 can also drive acquired drug resistance. Created in Biorender. Ge Zhang. (2025) https://app.biorender.com/illustrations/692c435a8836ddcb523362d8.

The role of DKK1 in canonical Wnt-dependent pathogenesis is widely supported by evidence from MM, HNSC, etc. In MM, the DKK1-mediated inhibition of osteoblast differentiation and therapy resistance is supported by extensive in vitro mechanistic assays and in vivo xenograft models, complemented by correlative patient data. The strength of these preclinical findings in MM also led to the development of the first-generation DKK1 antibodies. Detailed results from subsequent clinical trials will be discussed in the following sections. The multifaceted roles of DKK1 in cancer via canonical Wnt-signaling dependent mechanisms are summarized in Figure 1.

### 3.2. Canonical Wnt/β-Catenin-Independent Mechanisms

#### 3.2.1. Immune Modulation

In mismatch repair-deficient (dMMR) CRC, DKK1 was reported to serve as a critical determinant of resistance to PD-1 blockade. Clinical evidence showed a negative correlation between the elevated DKK1 levels, in both tumor tissue and serum, and the intratumoral CD8^+^ T cell infiltration. The results indicated that DKK1 might serve as a predictor of poor response to anti-PD-1 therapy. Mechanistically, DKK1 was shown to impair CD8^+^ T cell effector function by dysregulating the GSK3β/E2F1/T-bet signaling axis [56].

DKK1 was also reported to modulate the innate immune system within the TIME. In GC, tumoral DKK1 expression was reported to be associated with an immunosuppressive phenotype. In vitro assays suggested that DKK1 could drive tumor-associated macrophage (TAM) polarization toward a pro-tumorigenic state, which subsequently reduces the antitumor activity of CD8^+^ T cells and NK cells. Mechanistically, this process might be mediated by the binding of DKK1 to CKAP4 on the macrophage surface, which activates the downstream PI3K-AKT signaling pathway. Importantly, blocking DKK1 to reprogram TAMs has been shown to enhance the efficacy of PD-1 blockade in GC models [16].

Beyond macrophages, DKK1-CKAP4 interaction was also reported to drive neutrophil dysfunction in breast cancer. It was suggested that the interaction between DKK1 and CKAP4 could initiate the STAT6 axis, leading to the upregulation of CHI3L3. This process maintains neutrophils in an immature, immunosuppressive state that abrogates CD8^+^ T cell-mediated immunity. Furthermore, pharmacological inhibition of DKK1 could promote neutrophil maturation and sensitize bone metastases to immune checkpoint blockade, providing a novel strategy to overcome multi-lineage immunosuppression [14].

In addition, it was reported that DKK1 could inhibit the monitoring function of NK cells on tumors [57]. In breast cancer, studies showed that both systemic DKK1 (originating from the bone microenvironment) and local DKK1 (secreted by CAFs) could damage the activity of NK cells. Unlike the signaling activation observed in the myeloid cells, it was reported that DKK1 could inhibit the cytotoxicity of NK cells by weakening the phosphorylation of key effectors, including AKT, ERK, and S6 [58]. A similar mechanism was observed in metastatic castration-resistant prostate cancer (mCRPC). The biopsies from patients with high expression of DKK1 showed an accumulation of quiescent NK cells and a reduction in activated subgroups. Notably, the antitumor efficacy of the anti-DKK1 monoclonal antibody (DKN-01) was found to be largely dependent on NK cells, since in preclinical mouse models, its inhibitory effect disappeared following NK cell depletion [59].

Collectively, these findings suggested that DKK1 could serve as a multifunctional immunosuppressor that disrupts both innate and adaptive immune responses, indicating it as a promising target for overcoming immunotherapy resistance.

#### 3.2.2. Promoting Progression and Metastasis

Beyond contributing to the immunosuppression within the TIME, DKK1 was also reported to promote tumor progression through interacting with CKAP4. In vivo studies in immunodeficient mice showed that antibody-mediated blockade of the DKK1-CKAP4 interaction downregulated AKT signaling and attenuated xenograft tumor formation in a variety of malignancies, including esophageal cancer [60], as well as pancreatic and lung cancers [15]. In addition, CKAP4 has also been shown to be secreted in small extracellular vesicles (sEVs) from pancreatic ductal adenocarcinoma (PDAC) cells through a DKK1-dependent endocytic pathway. Therefore, it indicated that anti-CKAP4 monoclonal antibodies might exhibit dual potential as therapeutic agents targeting DKK1-CKAP4 signaling and as diagnostics for detecting circulating CKAP4 [61].

Unlike the CKAP4 axis, it was reported that, in prostate cancer, DKK1 could promote tumor growth and metastasis through the non-canonical Wnt/JNK signaling axis [62]. In addition, recent research showed that DKK1 knockout could lead to the re-expression of E-cadherin and its membrane co-localization with β-catenin, alongside the downregulation of the mesenchymal marker Cadherin-11. It indicated that DKK1 could promote migration not only through kinase signaling but also by modulating epithelial–mesenchymal transition (EMT) dynamics [63]. Collectively, these studies suggested that DKK1 could promote malignancy through highly complex and multifaceted pathways. It is urgent to carry out more in-depth research to fully unravel its distinct molecular mechanisms.

In esophageal adenocarcinoma, in vitro functional studies suggested that DKK1 expression is essential for maintaining malignant behaviors, including cell viability, proliferation, migration, and invasion. Intriguingly, these pro-tumorigenic effects are distinct from canonical Wnt signaling regulation, since neither DKK1 knockdown nor exogenous supplementation alters the transcriptional activity of β-catenin. Instead, evidence suggested that DKK1 can regulate downstream Akt phosphorylation [64]. However, the precise molecular mechanism between DKK1 and Akt activation is not clear and needs further investigation.

In HCC, the functional role of DKK1 appears complex. One report suggested that DKK1 could promote cell migration and invasion rather than proliferation. In this process, DKK1 was reported to upregulate β-catenin and Matrix Metalloproteinase 7 (MMP7), which is completely different from its typical mechanism of action as an inhibitor of Wnt/β-catenin signaling. Further clinical sample examination consistently showed that there was a positive correlation between DKK1 and β-catenin levels and tumor metastasis [65]. Conversely, recent studies indicated a broader pro-tumorigenic function for DKK1 in HCC. Experimental evidence suggested that DKK1 could promote not only migration and invasion but also proliferation. Its knockdown or antibody-mediated blockade effectively suppresses these phenotypes and inhibits tumor growth in vivo [9]. While collective evidence suggested the pro-oncogenic nature of DKK1 in HCC, the disparate signaling pathways controlling proliferation versus invasion remain to be fully elucidated.

Moreover, apart from its modulation of the immune landscape in HNSC, DKK1 was reported to exert direct intrinsic effects on tumor cells. It was reported that knockdown of DKK1 could impair cell viability and colony-forming capacity, although the precise underlying mechanisms remain unclear [49].

#### 3.2.3. Driving Angiogenesis

It was reported that the DKK1-CKAP4 axis could also play a pivotal role in remodeling the tumor vascular niche. In cholangiocarcinoma, DKK1 was found as a paracrine factor by binding to CKAP4 expressed on vascular endothelial cells (e.g., HUVECs and HHSECs). This interaction subsequently activates the PI3K-AKT signaling pathway, leading to PLVAP upregulation and subsequently enhancing the angiogenic potential of endothelial cells. These findings suggested that the DKK1-CKAP4-PI3K-PLVAP axis might represent a promising therapeutic target for anti-angiogenic intervention [66]. In HCC, DKK1 was also reported to contribute to tumor aggressiveness by stimulating angiogenesis [9]. Further mechanistic studies indicated that DKK1 could activate the VEGFR2-mediated mTOR/p70S6K signaling cascade [67].

#### 3.2.4. Fostering Therapy Resistance

Highlighting the complexity of DKK1 signaling, contemporaneous research suggested that DKK1 could also confer resistance to proteasome inhibitors through a distinct Wnt-independent mechanism in MM [55]. One report showed that BTZ-resistant MM cells exhibited significantly elevated DKK1 levels. Mechanistically, the binding of DKK1 to CKAP4 triggers the NF-κB signaling pathway by recruiting cullin-associated and neddylation-dissociated 1 (CAND1). This interaction could prevent CAND1 from inhibiting the assembly of the E3 ligase complex, thereby accelerating the ubiquitination and degradation of IκBα and sustaining NF-κB activation. Moreover, this resistance phenotype was reported to reinforce by a feedback loop in which NF-κB–driven interleukin-6 (IL-6) upregulated CKAP4 expression. Consequently, targeting the DKK1-CKAP4 axis might offer a therapeutic potential by restoring sensitivity to BTZ in MM [68].

In CRC, chemotherapeutic agents such as 5-fluorouracil and oxaliplatin was reported to trigger the secretion of DKK1 from CAFs via the activation of the MEK/ERK/p53 signaling axis. This chemotherapy-induced increase in DKK1 could foster resistance by promoting an immunosuppressive state. Specifically, it was reported that DKK1 could promote the secretion of immunosuppressive cytokines by CAFs, drive the infiltration of MDSCs, and restrict the recruitment of NK cells [40].

**Figure 2:**
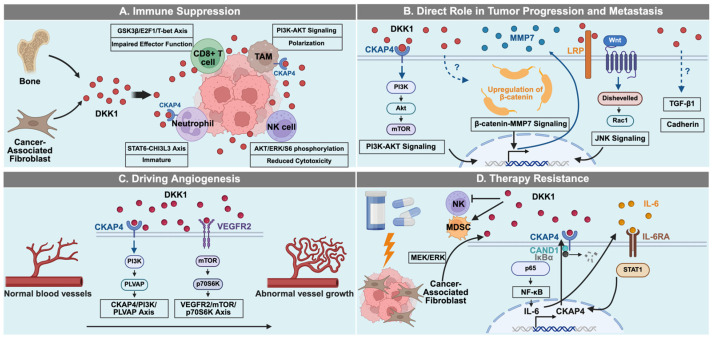
The multifaceted roles of DKK1 in cancer via non-canonical Wnt-signaling mechanisms. (**A**) Immune Suppression: DKK1 can recruit and sustain immature neutrophils and promote the polarization of TAMs. Concurrently, DKK1 can attenuate NK cell cytotoxicity and impair CD8^+^ T cell effector function. (**B**) Direct Role in Tumor Progression and Metastasis: DKK1 can drive tumorigenesis through distinct pathways. (**C**) Driving Angiogenesis: DKK1 can drive the transition from normal to pathological vasculature. (**D**) Therapy Resistance: DKK1 can orchestrate therapeutic resistance. Created in Biorender. Ge Zhang. (2025) https://app.biorender.com/illustrations/69293c97e7dbaeaf1b42df75.

Collectively, beyond its canonical role as a Wnt antagonist, DKK1 acts as a multimodal oncogenic regulator through non-canonical Wnt-independent mechanisms (Figure 2). High-quality in vitro assays and in vivo preclinical studies suggested its potential as a promising therapeutic target across a broad spectrum of cancer types, such as MM, esophageal adenocarcinoma, HCC, CRC, CRPC, PDAC, etc.

It is worth noting that the role of DKK1 in cancer depends on the specific biological background and tumor stage. This functional conversion may be regulated by the following factors. For example, the ratio of type of receptors (LRP and CAKP) on the cell surface may regulate the dominant pathway of DKK1. In addition, the genetic mutations in tumors may also play a role. For instance, in cancers where the Wnt signaling has been activated by mutations, DKK1 may not be able to inhibit Wnt signaling. Next, since DKK1 could play an important role in protecting cancer cells from immune system attacks, the component and distribution of TIME may also play a key role in this process. Therefore, more in-depth research is still needed to fully understand how these factors work together.

## 4. DKK1 as a Diagnostic and Therapeutic Target: Emerging Preclinical Strategies and Clinical Transitions

### 4.1. Therapeutic Monoclonal Antibodies

#### 4.1.1. BHQ880

BHQ880 represents a first-in-class, fully human monoclonal antibody (IgG1 isotype) specifically designed to target DKK1, and was the first agent of its kind to undergo clinical evaluation. In the context of MM and bone-metastatic solid tumors, the abnormally secreted DKK1 antagonizes the canonical Wnt signaling pathway. This disruption disrupts the osteoblast–osteoclast equilibrium, leading to osteolytic lesions.

BHQ880 exerts its therapeutic potential through a dual, environment-dependent mechanism. On the one hand, it was reported that BHQ880 could neutralize DKK1 to relieve the inhibition of Wnt signaling, thereby promoting osteoblast differentiation and restoring bone mineral density (BMD). On the other hand, although it was reported that BHQ880 showed no inhibitory effect on MM cells, it could indirectly suppress tumor proliferation by modulating the bone marrow microenvironment. Mechanistically, it could disrupt the adhesion between MM cells and bone marrow stromal cells (BMSCs), thereby inhibiting the secretion of IL-6, a key survival factor. The preclinical research of Fulciniti et al. evaluated this mechanism. In vivo studies showed that BHQ880 could significantly increase BMD while simultaneously impeding MM progression in a severe combined immunodeficiency (SCID)-hu murine model of human MM [69]. However, the anti-tumor efficacy of DKK1 inhibition appears to be different in different models. In another study using the syngeneic 5T2MM murine model, Heath et al. found that while BHQ880 could effectively prevent the loss of trabecular bone volume (BV/TV) and reduce osteoclast activity, it did not significantly alter overall tumor load [70]. The above preclinical data suggest that the bone-protective effect of DKK1 inhibition is stable, but its anti-tumor potential still needs to be further investigated.

At present, BHQ880 has been evaluated in a number of Phase I/II clinical trials, primarily focusing on high-risk smoldering multiple myeloma (SMM) and relapsed/refractory multiple myeloma (RRMM). In a Phase II trial for SMM, BHQ880 showed robust bone-modulating efficacy. High-sensitivity quantitative computed tomography (qCT) combined with finite element analysis showed that after 6 months of treatment, a significant 3% average increase was observed in vertebral strength (*p* = 0.002). The treatment also showed favorable safety, with no cases of osteonecrosis of the jaw (ONJ) reported [71]. However, research by Iyer et al. indicated that while BHQ880 combined with standard therapy was safe in patients with RRMM, it did not significantly improve the objective response rate (ORR) or reduce serum M-protein levels, the primary biomarker for monitoring MM progression [72].

Although BHQ880 effectively validated DKK1 as a bone regulation target, its research and development in MM has been basically stagnated due to the limited monotherapy efficacy compared to the current immunotherapy benchmarks. This indicates that although DKK1 inhibition exhibits advantage in restoring bone integrity, its future therapeutic potential may lie in targeting immune evasion within the TME of solid tumors.

#### 4.1.2. DKN-01

Therefore, the next generation of DKK1-neutralizing antibody, DKN-01, has been developed. Unlike its predecessors that focused on bone regulation, DKN-01 is being positioned as an immunomodulator for solid tumors, such as gastric and gastroesophageal junction (G/GEJ) cancers. A key structural feature of DKN-01 is its IgG4 isotype, which exhibits reduced antibody-dependent cellular cytotoxicity (ADCC) [57].

DKN-01 has shown a wide range of therapeutic potential in solid tumor therapy due to its ability to regulate the TIME. Preclinical studies utilizing a murine-derived alternative antibody (mDKN-01) suggested that DKK1 blockade could exert strong anti-tumor effects in melanoma and metastatic breast cancer models. Further mechanism studies suggested that this efficacy is dependent on an intact immune system and shows synergy with anti-PD-1 therapy [57,58]. In intrahepatic cholangiocarcinoma (iCCA) models, DKN-01 also showed anti-tumor activity. In the hydrostatic tail vein injection (HTVI) model, DKN-01 treatment significantly reduced tumor load (from 42.36% to 18.45%) and decreased tumor nodule count (from 104 to 58). In the KPP injury-induced model, tumor load was also reduced from 24.5% to 4.7% [73].

DKN-01 is undergoing Phase I/II clinical evaluation across diverse solid tumors (Table 2). In the DisTinGuish trial (Phase II Part A), DKN-01 combined with tislelizumab and CAPOX as first-line therapy for HER2-negative advanced gastric–esophageal adenocarcinoma (aGEA) achieved an impressive ORR of 73% with median PFS and OS of 11.3 and 19.5 months, respectively [10]. For second-line combination therapy in high-DKK1 GEA (DisTinGuish Part B), efficacy surpassed historical benchmarks with an ORR of 21.7% (31.8% in the PD-L1 ≥ 5% subgroup) and median OS of 8.2 months [74]. In the aforementioned clinical trial, DKK1 was used as a prospective biomarker for patient selection, and an H-score of ≥35 obtained via RNAscope was defined as “high DKK1 expression”. These findings suggested DKK1 as both a therapeutic target and a predictive biomarker for patient stratification in GEA. In a Phase I/II study of mCRPC, DKN-01 plus docetaxel achieved a 71% partial response rate and median rPFS of 5.7 months, though efficacy showed no clear correlation with DKK1 expression, suggesting a potentially broader mechanism of action in prostate cancer [75]. In contrast, in a Phase II basket study of recurrent endometrial cancer [42], efficacy was highly dependent on DKK1 expression levels: In this retrospective analysis, patients with an RNAscope H-score in the upper tertile (H-score ≥ 18) were defined as having high DKK1 expression. DKN-01 monotherapy demonstrated significantly superior ORR (25.0% vs. 0%), DCR (62.5% vs. 6.7%), and survival benefits (mPFS 4.3 vs. 1.8 months; mOS 11.0 vs. 8.2 months) in the high-DKK1 expression group compared to the low-expression group, suggesting promising clinical application prospects. The DeFianCe study (ClinicalTrials.gov, NCT05480306; registered on 29 July 2022) [76] is a Phase II, randomized, open-label, multicenter clinical trial evaluating the efficacy of sirexatamab (DKN-01) in combination with bevacizumab and chemotherapy in patients with advanced CRC. Results demonstrated that sirexatamab significantly improved clinical outcomes in two biologically well-defined exploratory populations: those with high DKK1 expression or those who had not previously received anti-VEGF therapy. Specifically, in patients with high expression of DKK1, the experimental arm showed a 32% higher ORR compared to the control arm, with PFS extended by 3.5 months and a trend toward prolonged OS. Among patients who had not received anti-VEGF therapy in the past, the experimental group demonstrated a 22% higher ORR and a 2.6-month-longer PFS. Although OS data were not yet mature, a favorable trend was observed. The study is still ongoing with continued follow-up, expected to further clarify the potential benefits of sirexatamab in the treatment of advanced colorectal cancer.

Furthermore, DKN-01 has demonstrated manageable toxicity profiles across various solid tumors. In the DisTinGuish trial for advanced gastric and gastroesophageal junction adenocarcinoma, the combination of DKN-01 and tislelizumab exhibited a manageable safety profile. In Part A (first-line treatment, in combination with the CAPOX regimen), common adverse events (AEs) were primarily low-grade, including nausea (72%), diarrhea (64%), and fatigue (60%); treatment-related adverse events (TRAEs) of Grade 3 or higher associated with DKN-01 remained relatively rare. In Part B (second-line treatment, without chemotherapy), the incidence of DKN-01-related adverse events was 40.4%, with fatigue (15.4%) and nausea (9.6%) being the most common signals. In clinical studies of recurrent endometrial cancer and mCRPC, DKN-01 demonstrated good safety and tolerability, whether as monotherapy or in combination with paclitaxel or docetaxel. Common AEs such as fatigue, nausea, and anemia were mostly Grade 1–2; in the mCRPC trial, no Grade ≥3 adverse events related to DKN-01 were observed, and the profile of serious adverse events (SAEs) in the combination therapy group was consistent with the known safety data of the chemotherapeutic agents, with no signs of additive toxicity caused by DKN-01.

In summary, DKN-01 represents a paradigm shift from bone protection to immune-oncology. However, the recent discontinuation of the global Phase III trial in first-line GC underscores the formidable challenge of outpacing the current Standard of Care (SoC). While DKN-01 is biologically active, achieving a statistically significant benefit over the now-standard PD-1 + chemotherapy regimen remains elusive in a large-scale, heterogeneous population. This outcome prompts people to re-evaluate the DKK1-targeted strategies, emphasizing the need for biomarker-driven stratification of patients. For example, identifying the therapeutic window where DKK1 inhibition could uniquely reverse immune evasion, particularly in PD-L1 low or cold tumors. In addition, DKK1 showed a strong clinical relevance, but the anti-tumor activity of DKK1 antibodies seem to need to be further improved, indicating that DKK1 may play other unrecognized non-canonical roles in oncogenesis in addition to the known immune regulation. In addition, the binding epitope of DKN-01 might be limited, so it may not be possible to block alternative downstream signaling pathways that directly drive cancer development. Hence, it suggests the need to develop next-generation therapeutics capable of more comprehensive pathway inhibition.

#### 4.1.3. Critical Considerations for Clinical Translation

Although BHQ880 and DKN-01 have provided a clinical proof of concept for targeting DKK1, the overall treatment benefits observed so far are still relatively limited. For BHQ880, its primary mechanism of action involves modulating the bone marrow microenvironment, but this microenvironment regulation alone may be not enough to induce significant tumor regression in MM. For DKN-01, although encouraging activity has been observed in specific subgroups, especially in tumors with high DKK1 expression and combined treatment regimens, these results were not enough to establish DKK1 inhibition as a widely effective strategy in unscreened patient groups. Therefore, DKK1 may be regarded as a mediator that depends on a specific situation rather than a widespread oncogenic driver. Current patient screening methods primarily rely on DKK1 expression H-scores assessed using rRNA-scope technology; however, optimal thresholds have not yet been uniformly standardized. Furthermore, intratumoral heterogeneity, temporal variability in DKK1 expression during treatment, and inconsistencies between primary and metastatic lesions may all reduce the predictive value of a single baseline biopsy. Consequently, while biomarker-guided enrichment may improve signal detection, it may also oversimplify the biological complexity of DKK1-driven tumors. Redundancy in signaling pathways may further limit clinical efficacy. DKK1 is involved not only in classical Wnt-related signaling but also in oncogenic signaling pathways, including those associated with CKAP4, as well as immune regulatory circuits within the tumor microenvironment. Consequently, when compensatory pathways remain active, extracellular neutralization of DKK1 alone may be insufficient to completely inhibit downstream pro-tumor signaling. Finally, significant interpatient heterogeneity further complicates clinical translation. The biological role of DKK1 is likely to vary depending on tumor type, disease stage, metastasis patterns, and prior treatment history. Furthermore, treatment efficacy may also depend on a variety of coexisting factors, such as immune rejection, myeloid-driven immunosuppression, prior anti-VEGF or anti-PD-1 therapy, and stroma-dependent factors. Taken together, these findings suggest that the future research and development of DKK1-targeted therapies requires more refined biomarker strategies, rational combination regimens, and clinical designs tailored to specific indications, rather than a one-size-fits-all, pan-cancer approach.

### 4.2. Other Emerging Modalities in Preclinical Studies

#### 4.2.1. Antibody-Drug Conjugates

Antibody-drug conjugates (ADCs) usually rely on sustained high-density antigen expression on the cell membrane and endocytosis for drug delivery. Therefore, DKK1, as a secretory protein, may be limited in its application as an ADC target. However, continuous in-depth study of DKK1 receptor network provides a new theoretical basis for its application as an ADC target. Kimura et al. have identified CKAP4 as a cell surface receptor for DKK1. Highly expressed in multiple tumor types, CKAP4 was reported to mediate the entry of the DKK1-CKAP4 complex into tumor cells via clathrin-dependent endocytosis, thereby activating the PI3K/AKT signaling pathway to promote tumor growth [15]. With the further elucidation of the DKK1-CKAP4-PI3K signaling pathway, the delivery of cytotoxic payloads via receptor-mediated internalization has emerged as a novel research direction.

#### 4.2.2. Aptamers

Aptamers are short single-stranded DNA or RNA oligonucleotides selected through the Systemic Evolution of Ligands by Exponential Enrichment (SELEX) technology. By folding into unique three-dimensional conformations, they bind to target molecules with remarkable specificity. Compared with traditional antibodies, aptamers have unique advantages, including superior thermal stability, low-cost chemical synthesis, and low immunogenicity [77,78]. Given the biomarker value of DKK1 in various diseases, nucleic acid aptamers, as novel molecular tools, have gained attention in the precise recognition and functional regulation of this protein.

In diagnostic applications, traditional immunoassays suggested DKK1’s potential in early HCC detection, achieving a sensitivity of 70.9% and a specificity of 90.5% [44]. Notably, different SELEX technical methods could affect the dynamic characteristics of the aptamers. Zhou et al. proposed a “slow dissociation SELEX” strategy, showing that only aptamers with slow dissociation rates could withstand multiple washing steps in ELISA assays. This novel DKK1 aptamer was reported to be a viable substitute for capture antibody in early HCC screening [79]. On the other hand, Shatunova et al. employed a combination DNA library design featuring a predefined purine/pyrimidine alternating pattern (YR pattern), significantly enhancing screening efficiency for chronic immunoinflammatory rheumatic diseases, such as axial spondyloarthritis (AxSpA). Through four rounds of selection, they obtained DK-series aptamers with affinities ranging from 1.3 to 3.7 nM [80]. These advancements indicate the potential of aptamers to transition from laboratory practice to next-generation diagnostic platforms.

In terms of therapeutic mechanisms, targeting the DKK1-CKAP4 non-canonical signaling axis represents a novel direction. The spl3c aptamer developed by Wei et al. was reported to not only block CKAP4-integrin interactions but also inhibit DKK1-CKAP4 interaction [81]. This mechanism could downregulate the PI3K/AKT signaling pathway and suppress actin cytoskeletal remodeling, thus inhibiting tumor growth and metastasis in bladder cancer cell models. Collectively, these findings indicate the potential of DKK1-targeted aptamers in treatment.

Although these preclinical results are encouraging, several key bottlenecks still need to be solved in the transition of DKK1-targeted aptamers from bench to bedside. On the one hand, the in vivo pharmacokinetics of oligonucleotides is still a major concern. Although aptamers exhibit low immunogenicity, their susceptibility to nuclease degradation and rapid renal clearance necessitates chemical modifications or encapsulation within nanocarriers to maintain therapeutic concentrations. On the other hand, the therapeutic synergy between aptamer-mediated non-canonical pathway inhibition (e.g., DKK1-CKAP4) and antibody-mediated immune modulation (e.g., DKK1-LRP5/6) warrants further investigation. A combinatorial approach or the development of bi-specific aptamer-antibody conjugates may potentially offer a more comprehensive neutralization of DKK1’s multifaceted roles.

#### 4.2.3. Small-Molecule Inhibitors

Current pharmacological approaches primarily focus on disrupting DKK1-receptor protein–protein interactions (PPIs), suppressing DKK1 expression, or modulating its epigenetic landscape.

NCI8642 acts as a specific inhibitor of DKK1-LRP6 interactions. Surface plasmon resonance (SPR) analysis showed that NCI8642 could bind to the LRP6 receptor with micromolar affinity (Kd ≈ 0.47 μM), effectively displacing DKK1 and restoring Wnt-β-catenin signaling [82].

Targeting upstream regulatory cascades may provide a new strategy for DKK1 inhibition. It was reported that Dorsomorphin could significantly downregulate the expression level of DKK1 mRNA and protein in breast cancer cells. However, the specific mechanism of action of Dorsomorphin on breast cancer cells still needs to be further investigated [83]. Furthermore, in the osteolytic prostate cancer models, the p38 MAPK signaling pathway has been identified as a key driver of DKK1 overexpression. The report pointed out that p38 small-molecule inhibitors, such as LY2228820 and SB202190, could significantly inhibit DKK1 expression, thus reversing its inhibitory effect on osteoblast differentiation [84].

However, simple expression inhibition or competitive inhibition often faces challenges such as incomplete signaling blocking or rapid compensatory upregulation. With the development of technique, future strategies may also shift to next-generation modalities, such as Proteolysis-Targeting Chimeras (PROTACs). This could induce the degradation of DKK1 or its receptor CKAP4, thus achieving more lasting downstream signal blocking, which may represent a promising future direction.

## 5. Challenges and Future Perspectives

**Figure 3:**
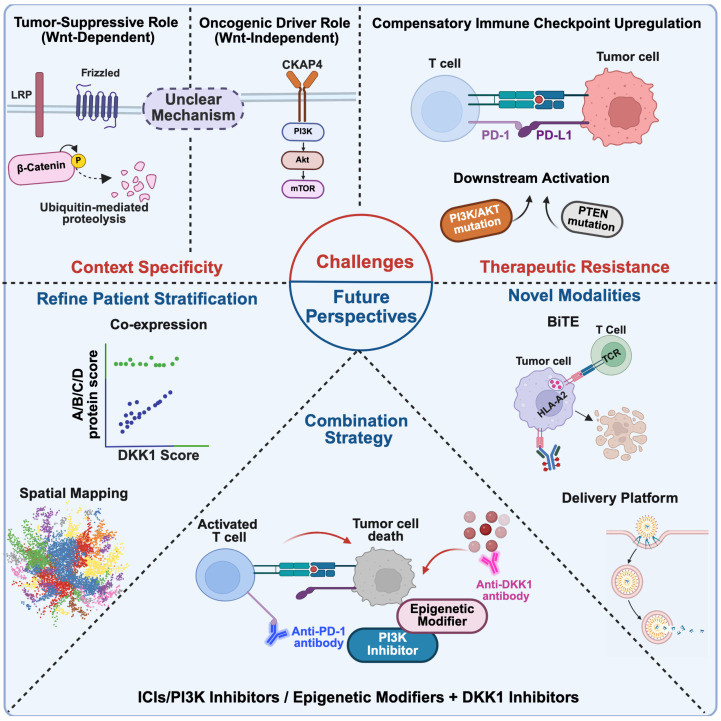
Challenges and future perspectives in DKK1-targeted therapy. Context Specificity: DKK1 could exhibit a complex functional role depending on the signaling context. Therapeutic Resistance: Resistance might arise from compensatory immune checkpoint upregulation or downstream pathway activation. Refine Patient Stratification: To improve therapeutic efficacy, patient selection might move beyond single biomarkers. Combination Strategy: Pairing anti-DKK1 antibodies with other treatments to synergistically improve therapeutic efficacy. Novel Modalities: Innovation in drug formats and optimization delivery forms are crucial for next-generation development. Created in Biorender. Ge Zhang. (2025) https://app.biorender.com/illustrations/69cb7fd8cd2605d8f4579a39.

The anti-tumor strategies targeting DKK1 showed potential application value in a variety of solid tumors. However, due to its functional heterogeneity in different tumor types and disease stages, patient selection becomes complicated because of reasons including lack of biomarkers, unclear efficacy prediction, unclear drug resistance, and so on (Figure 3).

### 5.1. Deciphering the Biological Dichotomy of DKK1: From Expression Profiles to Spatial Precision Medicine

DKK1 exhibits profound functional specificity. Therefore, accurate patient stratification is crucial. In a Phase II clinical study of advanced gynecological malignancies (primarily endometrial and ovarian cancers), Arend et al. found that baseline DKK1 expression level was positively correlated with activity of DKN-01 monotherapy or combination therapy. In the subgroup of patients with high expression of DKK1, DKN-01 could reshape the TIME more effectively, whereas minimal efficacy was observed in patients with low expression. This evidence suggested the necessity of adopting the DKK1 H-score as a companion diagnostic criterion [42]. However, a high H-score alone may not be enough to predict the outcome.

Looking forward, the integration of spatial transcriptomics and AI-driven pathological analysis may represent the next frontier direction to solve this complexity. Since DKK1 is a secretory protein, its functional influence depends on its spatial distribution range within the TME. By mapping the precise proximity of DKK1-secreting cells to tumor cells expressing CKAP4 and immune cells expressing LRP5/6, AI algorithms could be used to assist in quantifying the dominant signaling axis in a specific patient. This transition from bulk quantification to spatial precision medicine will be clearer to judge whether a malignancy is primarily driven by immune evasion or direct proliferation, thus guiding the final personalized treatment plan selection.

### 5.2. Physiological Safety Considerations of DKK1-Targeted Therapy

Targeted therapy against DKK1 raises safety concerns regarding the target. DKK1 acts as a physiological antagonist of the canonical Wnt/β-catenin signaling pathway in normal tissues; particularly in bone, it plays a role in osteoblast differentiation, bone remodeling, and fracture healing [2]. Theoretically, long-term systemic neutralization of DKK1 could disrupt bone and mineral homeostasis or impair repair processes during prolonged treatment. Studies have shown that reducing DKK1 gene dosage could increase bone formation and bone mass in vivo [85], and data indicated that dynamic changes in DKK1 are associated with the biology of fracture healing [86]. Beyond bone, DKK1 is also involved in intestinal epithelial homeostasis and injury responses, suggesting that chronic inhibition may produce broader, context-dependent effects in regenerative tissues [87]. Currently, risk mitigation and monitoring in clinical trial protocols for anti-DKK1 monoclonal antibodies primarily rely on standard clinical trial safety frameworks. These include the collection and grading of treatment-related adverse events (TEAEs) according to the NCI-CTCAE v5.0 criteria, the definition and protocol-based management algorithms for infusion reactions, serial hematological and biochemical testing, and dose interruption, reduction, or discontinuation measures in the event of toxicity. These protocols include laboratory monitoring related to bone homeostasis (e.g., calcium and phosphorus), and mCRPC protocols also explicitly require screening for serum 25-hydroxyvitamin D [75]. However, since most oncology trials are not designed to detect delayed or low-incidence skeletal-related outcomes, future studies with longer exposure periods that incorporate dedicated long-term bone safety assessments will help better define the therapeutic window for chronic systemic DKK1 inhibition.

### 5.3. Mechanisms of Resistance and Future Therapeutic Resilience

Although targeting DKK1 shows therapeutic potential, multidimensional resistance mechanisms should not be underestimated. These mechanisms may be broadly categorized into signaling bypass, compensatory immune regulation, and cellular plasticity.

Constitutive activation of downstream pathways may represent a key bypass mechanism. In tumors carrying gain-of-function mutations or overexpression of PIK3CA, AKT, or their downstream effectors (e.g., mTOR), the upstream blockade strategies targeting DKK1 may be of limited benefit. Hence, it may be helpful to combine downstream inhibitors. In addition, real-time monitoring of receptor expression (CKAP4 vs. LRP5/6) and downstream mutation status (e.g., PIK3CA or CTNNB1) via longitudinal biopsies or liquid biopsies will also be crucial for dynamic patient stratification.

Second, compensatory regulation within the TIME represents another potential mechanism of resistance. Haas et al. found that while monoclonal DKK1 blockade could restore innate immunity, tumor cells may establish new immune escape mechanisms by upregulating PD-L1 or other immune checkpoint molecules in preclinical models. This compensatory feedback loop may explain the limited efficacy of DKK1 inhibitors as monotherapies and underscores the necessity of combination treatment regimens [57].

Additionally, the plasticity and phenotypic transformation of tumor cells may also lead to intrinsic resistance to combination immunotherapy. Single-cell transcriptomics (scRNA-seq) studies in biliary tract cancers (BTCs) showed that the combination therapy with DKN-01 and anti-PD-1 antibody could induce malignant cells to undergo phenotypic shifts toward “neural-like” and “endothelial-like” states. These different cellular states showed inherent resistance to immune checkpoint blockade [88]. Hence, it is helpful to further explore the synergy between DKK1 inhibitors and epigenetic modifiers in the future to inhibit lineage-switching and maintain tumor sensitivity.

### 5.4. Next-Generation Therapeutic Platforms and Novel Drug Formats

At present, innovative therapeutic strategies are no longer limited to simple ligand neutralization. Novel monoclonal antibodies, such as DKK1-A2 mAbs, function as TCR-like antibodies that specifically recognize the DKK1-peptide-HLA-A2 complex. This mechanism could convert DKK1 from a soluble signaling ligand into a fixed cell-surface target. Furthermore, these antibodies provide a versatile scaffold for advanced engineering, such as bispecific T-cell engagers (BiTEs) and chimeric antigen receptor (CAR) therapies, which significantly improve their therapeutic potential and expand the database of precision medical tools for DKK1-positive malignancies [89].

In the field of cell therapy and vaccines, DKK1-based DNA vaccines showed dual effects, which could not only prevent the occurrence of tumors, but also regress established lesions in MM models. And the efficacy of these vaccines could be enhanced by CpG adjuvants [90]. However, the clinical translation of such vaccines should weigh the anti-tumor efficacy and the possibility of self-reaction in healthy tissues expressing DKK1.

In the field of nucleic acid therapeutics and novel delivery systems, multiple in vitro and in vivo studies showed the anti-tumor effects of siRNA-mediated targeted silencing of DKK1 on reducing tumor/inflammatory burden and alleviating tissue damage in a variety of models, including prostate cancer, atherosclerosis, rheumatoid arthritis, and multiple myeloma [63]. Although there is a still lack of clinical-grade nanoparticle formulations or LNP products specifically designed for DKK1, the RNA therapeutics field has established next-generation platforms utilizing lipid nanoparticles (LNPs), GalNAc conjugation, and various polymeric/inorganic nanocarriers for siRNA and mRNA delivery [91]. Studies involving cholesterol-modified DKK1-siRNA and DKK1-targeted ds-oligonucleotide self-assembled nanoparticles suggested that this target was suitable for integration with various nanodelivery strategies [92]. Despite this technical feasibility, a critical translational gap persists. For instance, optimizing LNPs for bone-marrow-specific delivery may maximize the therapeutic efficacy in MM while sparing other DKK1-dependent physiological processes. Ultimately, shifting from generic delivery to targeted precision nanomedicine will be essential for these next-generation platforms.

The therapeutic landscape of DKK1 is currently undergoing a transformative shift from a one-size-fits-all neutralizing approach to a multi-dimensional strategy.

## 6. Conclusions

In summary, DKK1 is a complex regulatory factor in cancer. Its roles are complex and diverse in different biological backgrounds and disease stages. It can not only block canonical Wnt/β-catenin signaling but also promote tumor growth and regulate immune escape through non-canonical pathways, such as the DKK1-CKAP4 axis. DKK1 was reported to be closely related to the prognosis of a variety of malignant tumors, indicating its application potential as a prognostic biomarker and therapeutic target. In the future, due to the high functional heterogeneity of DKK1 in a variety of malignancies, precision medicine targeting DKK1 may focus on integrating multi-dimensional patient stratification and shift from a one-size-fits-all neutralizing approach to a situation-based therapeutic strategy using novel modalities.

## Figures and Tables

**Table 1:** Pan-cancer expression landscape and prognostic significance of DKK1.

Cancer Type	Abbreviation	Expression (Tumor vs. Normal)	Prognostic Significance (High DKK1)	Hazard Ratio (95% CI)	Source IDs
Head & Neck Squamous Cell Carcinoma	HNSCC	Upregulated	Poor OS and DFS	2.48 (1.70–3.63)	[11]
Lung Adenocarcinoma	LUAD	Upregulated	Poor OS	-	[19]
Lung Squamous Cell	LUSC	Upregulated	Poor DFS	1.5 (-)	[18]
Stomach Adenocarcinoma	STAD	Upregulated	Poor OS	1.70 (1.19–2.44)	[17]
Esophageal Carcinoma	ESCA	Upregulated	Poor OS	2.23 (1.19–4.17)	[20]
Pancreatic Adenocarcinoma	PAAD	Upregulated	Poor OS	-	[21]
Liver Hepatocellular Carcinoma	LIHC	Upregulated	Poor OS	1.69 (1.12–2.54)	[22]
Cholangiocarcinoma	CHOL	Upregulated	Poor OS	1.90 (1.23–2.95)	[23]
Breast Cancer	BRCA	Upregulated	Poor OS	-	[24]
Prostate Cancer	PRAD	Upregulated	Poor OS	3.73 (1.44–9.66)	[25]
Multiple Myeloma	MM	Upregulated	Poor OS	-	[26]
Cervical Cancer	CESC	Upregulated	Poor OS	1.03 (-)	[27]
Ovarian Cancer	OV	Upregulated	Poor OS	4.40 (2.31–8.37)	[28]
Mesothelioma	MESO	Upregulated	-	-	[29]
Urothelial Carcinoma	UC	Upregulated	Poor DFS	2.44 (1.10–5.40)	[30]
Thyroid Cancer	THCA	Downregulated	Better Prognosis	2.48 (-)	[31]
Endometrial Cancer	UCEC	Downregulated	-	-	[32]
Colorectal Cancer	COAD/READ	Stage-Dependent	Poor OS	1.65 (1.22–2.24)	[33,34]

**Table 2:** Summary of clinical trial outcomes for DKN-01 in various solid tumors.

Cancer Type	Trial Phase/Name	Treatment Regimen	Patient Population	Key Efficacy Outcomes	Ref
aGEA	Phase II (DisTinGuish Part A)	DKN-01 + tislelizumab + CAPOX	1L, HER2-negative	ORR: 73%; mPFS: 11.3mo; mOS: 19.5mo	[10]
GEA	Phase II (DisTinGuish Part B)	DKN-01 + tislelizumab	2L, high-DKK1	ORR: 21.7% (31.8% in PD-L1 ≥ 5%); mOS: 8.2mo	[74]
mCRPC	Phase I/II	DKN-01 + docetaxel	Pre-treated	PR: 71%; mrPFS: 5.7mo (No DKK1 correlation)	[75]
Recurrent EC	Phase II (Basket study)	DKN-01 monotherapy	High-DKK1	ORR: 25.0%; DCR: 62.5%; mPFS: 4.3mo; mOS: 11.0mo	[42]
			Low-DKK1	ORR: 0%; DCR: 6.7%; mPFS: 1.8mo; mOS: 8.2mo	[42]
advanced CRC	Phase II	DKN-01 + bevacizumab + chemotherapy	High-DKK1	ORR: 48.0%; mPFS: 9.36mo	[76]
			Have not received anti-VEGF therapy	ORR: 55.1%; mPFS: 10.94mo	[76]

## Data Availability

No new data were created or analyzed in this study.

## Abbreviations

The following abbreviations are used in this manuscript:

DKK1	Dickkopf-1
CRDs	Conserved cysteine-rich domains
LRP5/6	Low-density lipoprotein receptor-related protein 5/6
CRC	Colorectal cancer
PTC	Papillary thyroid carcinoma
TCGA	The Cancer Genome Atlas
MM	Multiple myeloma
HCC	Hepatocellular carcinoma
GC	Gastric cancer
NSCLC	Non-small-cell lung cancer
OS	Overall survival
CKAP4	Cytoskeleton-associated protein 4
TIME	Tumor immune microenvironment
MDSCs	Myeloid-derived suppressor cells
NK	Natural killer
ICIs	Immune checkpoint inhibitors
CAF	Cancer-associated fibroblast
HNSC	Head and neck squamous cell carcinoma
TNBC	Triple-negative breast cancer
AFP	Alpha-fetoprotein
MESO	Mesothelioma
BTZ	Bortezomib
Hh	Hedgehog
TAM	Tumor-associated macrophage
mCRPC	Metastatic castration-resistant prostate cancer
sEVs	Small extracellular vesicles
PDAC	Pancreatic ductal adenocarcinoma
EMT	Epithelial–mesenchymal transition
MMP7	Matrix Metalloproteinase 7
CAND1	Cullin-associated and neddylation-dissociated 1
IL-6	Interleukin-6
BMD	Bone mineral density
BMSCs	Bone marrow stromal cells
SCID	Severe combined immunodeficiency
SMM	Smoldering multiple myeloma
RRMM	Relapsed/refractory multiple myeloma
qCT	Quantitative computed tomography
ONJ	Osteonecrosis of the jaw
ORR	Objective response rate
ADCC	Antibody-dependent cellular cytotoxicity
iCCA	Intrahepatic cholangiocarcinoma
HTVI	Hydrostatic tail vein injection
AEs	Adverse events
SoC	Standard of care
ADC	Antibody-drug conjugate
PPIs	Protein–protein interactions
SPR	Surface plasmon resonance
PROTACs	Proteolysis-Targeting Chimeras
BiTEs	Bispecific T-cell engagers
CAR	Chimeric antigen receptor
LNPs	Lipid nanoparticles
